# Influence of Prolonged Visual Display Terminal Use and Exercise on Physical and Mental Conditions of Internet Staff in Hangzhou, China

**DOI:** 10.3390/ijerph16101829

**Published:** 2019-05-23

**Authors:** Xiao Cheng, Mengna Song, Jingxia Kong, Xinglin Fang, Yuqing Ji, Meibian Zhang, Hongmei Wang

**Affiliations:** 1 Department of Social Medicine, Zhejiang University School of Medicine, Institute of Social Medicine and Family Medicine, Hangzhou 311000, China; xcheng92@163.com (X.C.); 21618445@zju.edu.cn (M.S.); kongjx_79@126.com (J.K.); 2 Zhejiang Provincial Center for Disease Control and Prevention, Hangzhou 311000, China; xlfang@cdc.zj.cn; 3 National Institute of Occupational Health and Poisoning Control, Beijing 100050, China; jiyq@niohp.china.cdc.cn

**Keywords:** visual display terminal, internet staff, exercise, physical health, mental health

## Abstract

To examine the effects of prolonged visual display terminal (VDT) working hours and exercise frequency on VDT-related symptoms, we recruited 944 Chinese internet staff for the study. A self-administered questionnaire survey was used to obtain the hours of daily VDT work, exercise frequency, and the physical and mental health of the participants. The daily VDT working time of participants was 8.7 hours. Musculoskeletal pain and eye complaints were prevalent, and the participants had poor mental health status. When daily VDT operation time was more than 11 hours, VDT-related symptoms, including backache (odds ratios (OR) = 3.59), wrist pain (OR = 1.88), hip pain (OR = 2.42), dry eyes (OR = 2.22), and ocular soreness (OR = 2.16) were more likely to occur, and an increased risk of serious occupational stress (OR = 6.75) and job burnout (OR = 2.66) was found in internet workers. Compared with those who never exercised, appropriate exercise frequency (three times per week) was helpful to relieve pain in the shoulders (OR = 0.28), neck (OR = 0.45), back (OR = 0.30), lower back (OR = 0.25), and wrists (OR = 0.38), as well as to prevent vision loss (OR = 0.33) and job burnout (OR = 0.42). Therefore, avoiding excessive VDT exposure and performing moderate exercise could protect the physical and mental health of internet staff from the adverse effects of VDT.

## 1. Introduction

Visual display terminals (VDTs) have been used in almost all institutions/organizations. In China, up to 95.2% of enterprises use computers in their daily work for a wide variety of vocational and/or non-vocational purposes [1]. Studies have shown that inappropriate use of VDT could jeopardize the visual, skin, and musculoskeletal systems and even the mental condition of workers [2,3,4,5,6,7]. Accordingly, these adverse effects make a significant contribution to reduced productivity at work. According to the Occupational Health Examination Regulation in China, VDT workers have been included in occupational health monitoring [8].

VDT operations are highly intensive in Internet companies, and staff were found to have impaired physical health [3,5,9] and mental health [10,11]. These factors were found to be relevant to VDT-related symptoms, such as the duration of occupation, daily working hours, distance from screen, breaks and rest, awkward postures, sedentary work, and high-intensity work [3,4,12]. The duration of VDT use is widely regarded as a key factor related to VDT-related symptoms [4,11,13,14]. Excessive overtime is common in most Chinese internet companies, especially those in prosperous and busy cities. However, few original studies have addressed the specific influence of long-time VDT work (more than 6 hours a day) [15].

Participating in regular to moderate exercise could be beneficial for physical health and emotional well-being [16,17]. Muscle tension and insufficient blood supply caused by sedentary work often leads to musculoskeletal pain [18]. Performing exercise not only strengthens muscles and improves physical status, it also alleviates stress and anxiety by activating the endocrine system [19]. It can relax eye muscles and improve optical adjusting functions, eventually preventing ocular disease. Therefore, in some studies, physical activity was accepted as an intervention in the management of body pain and mood disorder in the workplace [20,21,22,23]. Although a positive effect of physical activity on depression and anxiety was widely accepted [24,25], the effect on occupational stress remains controversial. Some studies indicated that exercising helped cope with perceived stress [24,26], while evidence also suggests that occupational stress of enterprise staff was not associated with leisure sports [27].

Our study aimed to describe the prevalence of VDT-related symptoms and their relationships with prolonged VDT working time among internet staff in Hangzhou. We also examined whether exercising could benefit workers in preventing or relieving such symptoms, especially occupational stress and job burnout.

## 2. Materials and Methods

### 2.1. Study Population

The type of businesses that participants were engaged in included design, telecommunications, and technology services. The inclusion criteria for participants were employees who had worked in their current position for at least 6 months and were without recent mental disease or psychotropic medication. Eligible VDT workers (technical staff, managers, sellers, etc.) were recruited from three internet companies in Hangzhou, China, by convenience sampling with the help of enterprise leaders. Each participant gave written informed consent and filled in the questionnaire on the spot.

### 2.2. Study Instrument

This study was part of a cross-sectional survey conducted in 2016. The entire survey tool consisted of a VDT work and health questionnaire, and a quality of life instrument (a Chinese version of the SF-36). Results on the quality of life of VDT workers has been published elsewhere [28]. The VDT work and health questionnaire included demographic data, VDT usage-related information, physical activity, and health conditions, which were analyzed in the current study.

#### 2.2.1. Demographic Data

Social-demographic characteristics included age, gender, marital status, education level, and annual household income per capita.

#### 2.2.2. VDT Usage-Related Information

Daily VDT working time was assessed using an open question: “On average, how many hours do you spend with your screen on work on each working day (including overtime)?”

Daily VDT leisure time was assessed using a multiple choice question: “On average, how many hours do you spend in front of a screen during leisure time in each working day?” Six options were provided: <2 h, 2–4 h, 4–6 h, 6–8 h, 8–10 h, ≥10 h.

#### 2.2.3. Physical Activity

Exercise frequency (never, 1–3 times per month, 1–2 times per week, 3 times or more per week) was measured using the question: “Do you perform any exercise?” Exercising for more than 30 minutes was recommended for effective health promotion; therefore, in this survey exercise was defined as physical activity lasting for more than 30 minutes for the purpose of exercising [29].

#### 2.2.4. Health Condition

Questions on symptoms of musculoskeletal pain were adapted from a valid Occupational Musculoskeletal Disorders Questionnaire [30], and some symptoms specific to VDT workers were selected, such as ache or pain in the shoulders, neck, back, lower back, wrists, and hip. Specific body pain was queried using the question: “Have you experienced pain or discomfort of the following body parts in the past six months (yes/no)?”

Eye discomfort included reduced vision, dry eyes, puffy eyes, ocular soreness, and eye irritation (yes/no).

Perceived occupational stress was valued using a scale from 0 (no pressure) to 10 (greatest pressure), where respondents selected a number to suggest their general occupational stress.

Job burnout was assessed using a single item: “The frequency of feeling *I’m tired of my job*” and the options were “0 (never),” “1 (several times per year),” “2 (once a month),” “3 (several times per month),” “4 (once a week),” “5 (several times per week),” and “6 (every day).”

A pilot study was conducted to examine the relevance, importance, comprehensibility, suitability, and potential redundancy of questionnaire items before the formal questionnaire was finalized. The language of this questionnaire was Chinese.

### 2.3. Data Collection

The respondents were asked to fill in the paper version after the study coordinators explained the instructions and requirements. All questionnaires were self-administered with minimal supervision by the study coordinators. It took approximately half an hour to complete the survey.

### 2.4. Statistical Analysis

Descriptive data was presented as percentages or as mean and standard deviation (SD). For further analysis, participants were categorized into two age groups (<30 years and ≥30 years) by the mean. Based on previous studies, the criteria for long-time VDT work was ≥6 h [15]. One standard deviation above the mean VDT working hours in our study (11 h) was considered excessive VDT exposure in daily work. Therefore, the participants were divided into three VDT working time groups (<6 h, 6–11 h, and ≥11 h). Outcome variables were self-reported musculoskeletal pain, eye discomfort, occupational stress, and job burnout. The presence of discomfort in different body parts were denoted as binary variables (yes or no). Perceived work stress and job burnout of participants were classified into subgroups on severity of mental disorders through expert consultation: (1) mild work stress (0–3), moderate work stress (4–7), and severe work stress (8–10); (2) no/mild job burnout (0–2: subjects feeling burnout once a month or fewer) and moderate/severe job burnout (3–6: subjects feeling burnout more than once a month).

The chi-square test was used to examine physical and mental differences in proportions of young (<30 years old) or male participants among different durations of daily VDT use and exercise frequency. The significance of the overall trend was tested using linear-by-linear association. Logistic regressions were performed to estimate the odds ratios (OR) and 95% confidence intervals (95% CI) for each outcome, with VDT working hours <6 h/day and never exercising as the reference, and adjustment for age, gender, and VDT time outside of work. Tests of trends in odds ratios across daily VDT working time and exercise frequency were calculated by recoding exposure levels as ordinal variables in the regression model. All analyses were conducted by Statistical Package for Social Sciences (SPSS) software, version 21, and a *p* value ≤0.05 was considered statistically significant.

## 3. Results

Of the 944 eligible VDT workers who participated in the survey, 915 participants were included in the final analysis. Among the 29 excluded questionnaires, 27 questionnaires were deleted for missing at least one SF-36 scale score, and two questionnaires were deleted for inconsistent information between two questionnaire datasets.

The demographic characteristics of participants are summarized in Table 1. More than half of the participants (58.0%) were younger than 30 years old, and there were more men (57.9%) than women. The mean work duration of the 915 participants in their current position was 48.3 months. Approximately two fifths of participants were exposed to VDT for more than 4 hours outside of work. For all participants, the mean working time per day with a computer was 8.7 hours. The majority of participants’ (81.5%) daily VDT working time was between 6–11 hours, while only 11.8% exercised three times or more per week. About four fifths of participants reported various musculoskeletal pain, with 56.8% in the neck, 48.9% in the shoulders, and 43.3% in the lower back. Eye irritation (50.3%), reduced vision (44.0%), and dry eyes (41.3%) were the most common eye complaints (85.0%). The majority (91.1%) of participants felt moderate or severe occupational stress and 39.1% had a feeling of job burnout more than once a month.

There was no significant difference in the proportions of young (<30 years old) or male participants for different VDT working times. The prevalence of selected health conditions in different working times are presented in Table 2. With the increase of daily VDT work, the proportion of participants reporting physical discomfort, such as backache, wrist pain, hip pain, dry eyes, and ocular soreness, also significantly increased (*p*-trend < 0.05). Those who operated VDTs for a longer time were more likely to have serious occupational stress and job burnout (*p*-trend ≤ 0.001).

Table 3 shows the number of participants (%) with demographic or health conditions among different exercise frequencies. The proportion of men decreased with increased exercise frequency, which means female participants tended to exercise more than male participants (*p* = 0.031). As exercise frequency increased, the prevalence of musculoskeletal pain were shown to be significantly lower in participants’ shoulders, neck, back, lower back, and wrists (*p*-trend < 0.05). About half of the people who never exercised had decreased vision and burnout feelings, which could be improved by engaging in more physical activities (*p*-trend < 0.05). No significant association between age group, perceived occupational stress, and exercise frequency was found.

The results of the logistic regression analysis adjusted by age, gender, and VDT time outside of work in all participants with each health condition per the dependent variables are given in Table 4 and Table 5. It was found that longer VDT working time indicated a significantly increased risk for physical symptoms such as backache, wrist pain, hip pain, dry eyes, ocular soreness, and job burnout (*p*-trend < 0.05). When the operation time exceeded 11 hours, VDT workers were increasingly susceptible to back pain (OR = 3.59), dry eyes (OR = 2.22), ocular soreness (OR = 2.16), and job burnout (OR = 2.66). The likelihoods of elevated occupational stress among staff having 6–11 hours and above VDT work were 1.82 and 6.75 times, respectively (all *p* < 0.05), compared with those having less than 6 hours VDT work per day. For participants who never exercised, doing exercises more often was a protective factor for musculoskeletal pain, reduced vision, and job burnout (*p*-trend < 0.05). Those who exercised at least three times a week had a much lower risk of physical and mental symptoms than other groups. Any significant association between exercise frequency and work stress was not observed.

## 4. Discussion

This study evaluated the adverse effects of excessive VDT use on physical and mental conditions among internet employees. It was found that prolonged working time had a significantly increased risk of backache, wrist pain, hip pain, dry eyes, ocular soreness, job burnout, and high occupational stress. Participating in exercise more than three times a week could significantly reduce health impairment of physical and mental conditions, such as musculoskeletal pain, reduced vision, and job burnout.

### 4.1. Prolonged VDT Work is a Health Hazard to Internet Staff

In our study, employees had an average VDT working time of 8.7 ± 2.2 hours per day, which was generally longer than the standard working hours (8 hours) in China [31]. With increasing daily VDT working time, employees reported worse musculoskeletal pain and eye discomfort, in addition to work pressure and job burnout. Similar results for the association between physical status, psychological status, and VDT operation time have been reported in previous studies [3,4,11].

Participants in our study were prone to report symptoms of discomfort in their back, wrists, hips, and eyes with longer working time. Employees who worked 6 to 11 hours had a comparable risk of having physical symptoms compared with those who worked less than 6 hours, while employees who worked at least 11 hours were more likely to have physical symptoms. VDT operators with awkward posture (e.g., slouching forward) or inappropriate workstation setup (e.g., placing the keyboard too low) would result in stress in many body regions [32]. If the condition continued for a long time, related muscles would be overstretched and cannot relax sufficiently, leading to musculoskeletal disorder. In contrast with previous studies [4,32], no significant difference was found in the incidence of neck pain among groups with different working times. This phenomenon is most likely related to the duration of VDT operation in our study, which was much longer and led to neck discomfort being commonly reported (56.8%). Other highly prevalent conditions, including shoulder pain (48.9%) and lower back pain (43.3%), were not associated with operation time either. Different body parts were subject to various VDT operation times. A daily working time of less than 6 hours may cause pain in the shoulders, neck, and lower back, while backache, wrist pain, and hip pain might occur with prolonged VDT operation daily. Thus, VDT users should be advised to take regular breaks and rest when early signs occur, in order to avoid any possible further injuries.

Higher risk of dry eye disease and eye soreness for longer VDT workers was found in our study, in accordance with other studies [2,3,14,33]. A certain blinking frequency is necessary to make tears spread across the corneal surface and keep eyes moist. Staring at a screen for hours could reduce the spontaneous blink rate, leading to unstable tear film and even dry eyes [34]. Significant eye discomfort related to VDT use should not be underestimated. With increasing VDT working time, VDT workers need to take appropriate measures, such as taking periodic breaks to prevent or to relieve eye symptoms.

In our study, over 90% of internet staff reported moderate or severe occupational stress, and nearly 40% of participants had feelings of burnout more than once a month. VDT workers reported higher levels of anxiety, depression, occupational stress, and job burnout [35,36]. Long working hours partly reflect heavy workload, high demands, and greater responsibilities, which always lead to higher occupational stress [37]. VDT operators often felt passive and monotonous and had high work demands, giving rise to boredom and stress [38]. In our study, among employees who were exposed to VDT at work for greater than 11 hours a day, the risk of elevated job stress was about six times greater than that of less than 6 hours. Therefore, it is urgent to pay more attention to the adverse effects of extended VDT working hours on employees’ mental health. The prevalence of job burnout is high, possibly due to overwork and long-term stressful situations, which are common in internet enterprises [39,40]. Age characteristics of participants could be another cause, since young workers generally had high levels of job burnout [41].

### 4.2. Greater Exercise Could Improve Physical and Mental Conditions of Internet Staff

In our study, only 11.8% of participants were exercising more than three times a week, indicating poor exercise behaviors. Physical activities were more common among female VDT workers in this study, which was inconsistent with other findings [42,43]. A heavier workload in male workers than in female counterparts in internet enterprises may be a possible cause of male workers’ diminished motivation to engage in sports [44].

Along with increased frequency of physical activity, the prevalence of musculoskeletal pain and fading eyesight decreased. The discomfort, stiffness, and pain in one’s neck, back, and shoulders may be alleviated by daily exercise [45,46]. Compared with those who never exercised, even low-frequency exercise (one to three times a month) could significantly alleviate pain in several body parts, such as the shoulders, back, and wrists. In order to prevent neck pain, lower back pain, and reduced vision, exercise should be performed at least three times a week. Therefore, varied and adequate exercise can be regarded as effective prevention and treatment for physical discomfort [21,23,47].

Although exercise helps prevent vision loss, it does not prevent other forms of eye discomfort. In accordance with a previous study [3], we did not find any significant relationship between exercise frequency and dry eyes, as the brightness of screens and angle of gaze played a vital role in most vision symptoms.

Regular exercise is regarded as an effective way to improve mental health, as it can increase self-confidence and lower symptoms associated with depression and anxiety [48,49]. A negative correlation was found between burnout feelings and exercise frequency, consistent with other findings [20,22]. Physical activities may eliminate negative emotion and may also make people stay enthusiastic [50]. Moreover, employees who exercised more than three times a week were at a much lower risk of job burnout, in accordance with the recommended exercise frequency [51,52]. However, there was no significant correlation between exercise frequency and occupational stress in this study. The relationship between exercise and occupational stress measured simultaneously using a cross-sectional survey is complicated, and cause and effect are inherently ambiguous. Physical activity relieves occupational stress by improving cognition and coping strategies [53,54]; in a similar way, it increases positive expectations and reduces symptoms of depression and anxiety. However, unlike the restriction of activity by enduring depression, some people may do more exercise when they are stressed [55]. Further longitudinal studies are needed to draw a causal inference.

### 4.3. Strength and Limitation

Our study examined the effects of excessive VDT operation (more than 11 hours a day) on the physical and mental health of internet staff, which currently has little available evidence. We also tried to understand whether moderate exercise (more than three times per week) had any effect on their psychological status. These findings could help to protect internet staff from the adverse effects associated with VDT work.

The main limitations of this study included its cross-sectional design and measurement. Longitudinal research is needed for causality. Single items rather than standardized instruments for measuring occupational stress and job burnout may cause bias. The lack of measurement of daily leisure time, breaks, and rest during the workday, and the exact exercise duration and frequency may also influence internal validity. Eligible VDT workers were recruited with the help of enterprise leaders; however, the number of invited staff was not available from the enterprises. Moreover, the participants were from three internet companies in Hangzhou, China, and are not representative of the target population in other areas. Therefore, caution is recommended if the results were extended to populations of different regions in this country.

## 5. Conclusions

VDT operation time was generally prolonged among internet employees in Hangzhou, China, and a variety of physical symptoms were reported, as well as serious occupational stress and job burnout. An increased risk of physical symptoms was found in internet workers with excessive VDT work (more than 11 hours per day). Also, the mental health of staff was impaired by overwork. Regular physical activity could alleviate some VDT-related physical symptoms and avoid job burnout.

## Figures and Tables

**Table 1:** Characteristics of the study participants.

Characteristics	N^a^	%
*Age (years)*		
<30	527	58.0
≥30	382	42.0
*Gender*		
Men	529	57.9
Women	384	42.1
*Daily visual display terminal (VDT) use outside of work*		
<2h	203	22.4
2–4h	346	38.2
≥4h	356	39.4
*Daily VDT use at work*		
<6h	70	7.7
6–11h	745	81.5
≥11h	99	10.8
≥*30 minute exercise frequency*		
Never	82	9.1
1–3 times per month	411	45.4
1–2 times per week	306	33.8
3 times or more per week	107	11.8
*Musculoskeletal pain*		
Shoulders	447	48.9
Neck	520	56.8
Back	296	32.3
Lower back	396	43.3
Wrists	161	17.6
Hips	110	12.0
*Eye discomfort*		
Reduced vision	403	44.0
Dry eyes	378	41.3
Puffy eyes	207	22.6
Ocular soreness	179	19.6
Eye irritation	460	50.3
*Occupational stress*		
Mild (0–3)	80	8.7
Moderate (4–7)	582	63.6
Severe (8–10)	252	27.5
*Job burnout*		
No/mild (0–2)	557	60.9
Moderate/severe (3–6)	358	39.1

a. Sample sizes within characteristics may not sum to *n* = 915 due to missing values.

**Table 2:** Number of participants (%) with health conditions among different durations of daily VDT use.

	Daily VDT Use			
	<6h (*n* = 70)	6–11h (*n* = 745)	≥11h (*n* = 99)	*p* Value
*Age <30 years*	42 (60.0)	422 (57.0)	63 (64.3)	0.369 ^1^
*Men*	40 (57.1)	437 (58.7)	52 (53.1)	0.558 ^1^
*Musculoskeletal pain*				
Back	14 (20)	236 (31.7)	46 (46.4)	<0.001 ^2^
Wrists	11 (15.7)	123 (16.5)	27 (27.2)	0.029 ^2^
Hips	6 (8.6)	86 (11.5)	18 (18.2)	0.039 ^2^
*Eye discomfort*				
Dry eyes	23 (32.9)	303 (40.7)	52 (52.5)	0.008 ^2^
Ocular soreness	12 (17.1)	136 (18.3)	31 (31.3)	0.01 ^2^
*Occupational stress*				
Slight (0–3)	14 (20.0)	64 (8.6)	2 (2.0)	<0.001 ^2^
Moderate (4–7)	42 (60.0)	497 (66.7)	43 (43.4)	
Heavy (8–10)	14 (20.0)	184 (24.7)	54 (54.5)	
*Job burnout*	22 (31.4)	282 (37.9)	54 (54.5)	0.001 ^2^

^1^ Chi-square test; ^2^ Linear-by-linear association.

**Table 3:** Number of participants (%) with demographic or health conditions among different exercise frequencies.

	Exercise Frequency				*p* Value
	Never (*n* = 82)	1–3 Times Per Month (*n* = 411)	1–2 Times Per Week (*n* = 306)	3 Times or More Per Week (*n* = 107)	
*Age <30 years*	44 (53.7)	249 (61.0)	172 (56.8)	57 (53.3)	0.342 ^1^
*Men*	54 (65.9)	243 (59.1)	178 (58.2)	49 (45.8)	0.031 ^1^
*Musculoskeletal pain*					
Shoulders	53 (64.6)	206 (50.1)	152 (49.7)	35 (32.7)	0.001 ^2^
Neck	54 (65.9)	241 (58.6)	176 (57.5)	48 (44.9)	0.023 ^2^
Back	38 (46.3)	140 (34.1)	95 (31.0)	22 (20.6)	0.001 ^2^
Lower back	47 (57.3)	193 (47.0)	127 (41.5)	28 (26.2)	<0.001 ^2^
Wrists	23 (28.0)	73 (17.8)	50 (16.3)	14 (13.1)	0.024 ^2^
*Eye discomfort*					
Reduced vision	43 (52.4)	193 (47.0)	138 (45.1)	29 (27.1)	0.001 ^2^
*Job burnout*	40 (48.8)	162 (39.4)	125 (40.8)	30 (28.0)	0.022 ^2^

^1^ Chi-square test; ^2^ Linear-by-linear association.

**Table 4:** Age-, gender-, and VDT time out of work-adjusted odds ratios (OR) and 95% confidence intervals (95% CI) of the duration of daily VDT use for selected health conditions.

	OR (95%CI)			
	<6h (*n* = 70)	6–11h (*n* = 745)	≥11h (*n* = 99)	*p*-Trend
*Musculoskeletal pain*				
Back	1.0	1.75 (0.94–3.24)	3.59 (1.74–7.41) ^1^	<0.001 ^2^
Wrists	1.0	0.97 (0.49–1.91)	1.88 (0.85–4.16)	0.042 ^2^
Hips	1.0	1.31 (0.55–3.14)	2.42 (0.90–6.52)	0.034 ^2^
*Eye discomfort*				
Dry eyes	1.0	1.42 (0.84–2.39)	2.22 (1.17–4.20) ^1^	0.011 ^2^
Ocular soreness	1.0	1.10 (0.57–2.10)	2.16 (1.01–4.61) ^1^	0.015 ^2^
*Occupational stress*	1.0	1.86 (1.09–3.15) ^1^	6.75 (3.52–12.93) ^1^	<0.001 ^3^
*Job burnout*	1.0	1.34 (0.79–2.27)	2.66 (1.39–5.085) ^1^	0.001 ^2^

^1^*p* < 0.05; ^2^ Binary logistic regression; ^3^ Ordinal logistic regression.

**Table 5:** Age-, gender-, and VDT time out of work-adjusted odds ratios (OR) and 95% confidence intervals (95% CI) of exercise frequency for selected health conditions.

	OR (95%CI)				
	Never (n = 82)	1–3 Times Per Month (n = 411)	1–2 Times Per Week (n = 306)	3 Times or More Per Week (n = 107)	*p*-Trend
*Musculoskeletal pain*					
Shoulders	1.0	0.57 (0.34–0.93) ^1^	0.59 (0.35–0.99) ^1^	0.28 (0.15–0.52) ^1^	0.001 ^2^
Neck	1.0	0.77 (0.47–1.28)	0.79 (0.47–1.32)	0.45 (0.24–0.82) ^1^	0.021 ^2^
Back	1.0	0.60 (0.37–0.98) ^1^	0.55 (0.33–0.92) ^1^	0.30 (0.16–0.57) ^1^	0.001 ^2^
Lower back	1.0	0.66 (0.41–1.09)	0.53 (0.32–0.88) ^1^	0.25 (0.13–0.47) ^1^	<0.001 ^2^
Wrists	1.0	0.53 (0.31–0.93) ^1^	0.50 (0.28–0.90) ^1^	0.38 (0.18–0.81) ^1^	0.024 ^2^
*Eye discomfort*					
Reduced vision	1.0	0.80 (0.49–1.28)	0.74 (0.45–1.21)	0.33 (0.18–0.61) ^1^	0.001 ^2^
*Job burnout*	1.0	0.67 (0.41–1.08)	0.71 (0.43–1.17)	0.42 (0.23–0.77) ^1^	0.028 ^2^

^1^*p* < 0.05; ^2^ Binary logistic regression.
